# Development of an Objective Structured Clinical Examination for Assessment of Clinical Skills in an Emergency Medicine Clerkship

**DOI:** 10.5811/westjem.2015.9.27307

**Published:** 2015-10-22

**Authors:** Sharon Bord, Rodica Retezar, Pamela McCann, Julianna Jung

**Affiliations:** Johns Hopkins University School of Medicine, Department of Emergency Medicine, Baltimore, Maryland

## INTRODUCTION

Assessment of medical students in their emergency medicine (EM) clerkship is often based on clinical shift evaluations and written examinations. Clinical evaluations offer some insight into students’ ability to apply knowledge to clinical problems, but are notoriously unreliable, with score variance that may be driven as much by error as by actual student performance.^1^–^6^ Clinical evaluations are also limited by the unpredictability of pathology in emergency department (ED) patients, and by patient safety considerations that prevent students from independently managing patients, especially those with high-acuity conditions. Additionally, there is evidence that the basic skills of history and physical exam are rarely observed by faculty members, and the feedback they receive on these domains is limited.^7^–^9^ These factors hinder EM educators in their effort to objectively assess students’ progress relative to clerkship objectives, particularly those that pertain to emergent care.

The objective structured clinical exam (OSCE) is one potential solution to these problems. Described in 1975 by Harden et al, an OSCE is designed to assess the clinical competence of medical trainees through direct observation of skill performance in a variety of stations.^10^ OSCEs have been widely adopted in medical education and in other health professions.^11^–^13^ These exams are viewed as a valuable form of clinical assessment due to their demonstrated reliability and inherent flexibility for assessing a wide variety of knowledge application and skills.^11^

In EM, OSCEs have been used mainly in postgraduate medical education to assess resident communication skills and clinical performance.^14^,^15^ One OSCE for interns was shown to accurately predict future clinical performance scores.^12^ A recently published evaluation of an EM OSCE for medical students demonstrated validity evidence supporting this method of assessment.^16^ These studies suggest that OSCEs can be used effectively in EM education, and yield valid assessment data.

## OBJECTIVE

Our primary goal was to develop an OSCE that would assess whether students have not only acquired essential knowledge during their EM clerkship, but are also able to synthesize this knowledge into a management plan and perform key critical actions in emergency situations. EM is a required core clerkship for all students at our institution, and occupies an essential place our medical student curriculum. While many students will not pursue careers in EM, all will confront emergencies throughout their careers, and emergency management skills are vital for all physicians. Rigorous assessment will allow students to appropriately focus their future learning to improve their skills in this arena, it will facilitate curricular improvements by giving educators insight into common errors and misconceptions, and it will permit documentation of student competency.

## CURRICULAR DESIGN

We designed our OSCE for use in the required core clerkship in EM, which is taken by students ranging from the final quarter of the MS-2 year to the third quarter of the MS-4 year. The exam is fundamentally criterion-referenced, in that all material covered in the exam is explicitly taught during the clerkship, and it is our expectation that all students will “pass.” However, as is the case with many assessments in medical education, there is an element of norm-referencing as well. While it was our hope that every student will demonstrate at least minimal competency, we expected to see a wide range of exam performance based on variation in student knowledge and abilities, and we sought to capture that range when developing the exam.

We developed the OSCE based on the EM Milestones, with the goal of assessing the majority of the clinically-oriented competencies described in that framework. The milestones that are assessed with exam include emergency stabilization, performance of history and physical exam, diagnostic skills, diagnosis, pharmacotherapy, airway management, observation and reassessment, goal directed focused ultrasound, professional values and patient centered care (Table 1). The exam consists of three stations: two manikin-based simulations and one standardized patient encounter. These scenarios were developed and vetted by a core group of undergraduate medical educators in the department of EM at our institution. Specific cases and complaints were chosen in order to highlight challenges and topics that are unique to the field of EM and represented in the Milestones. All cases were designed to assess the student’s ability to both assess and manage critical illness independently.

The cases depict the following diagnoses: massive pulmonary embolus, intra-abdominal bleeding after blunt trauma, and poly-substance overdose with airway compromise. Each case has two phases: a stable “assessment” phase, and an unstable “treatment” phase. During the student’s evaluation, all patients become unstable, necessitating rapid resuscitation. Cases are described below, and key checklist items are summarized and mapped to Milestones in Table 1:

Case 1: Massive pulmonary embolus. In this manikin scenario, a patient presents three days following total knee replacement with acute chest pain and dyspnea. The patient develops pulseless electrical activity that later degenerates to ventricular fibrillation. Students are expected to perform needed assessment, order appropriate diagnostics for pulmonary embolus, and provide advanced cardiovascular life support interventions for cardiac arrest.

Case 2: Blunt abdominal trauma. In this standardized patient encounter, a young female patient is assaulted and presents with head, neck, and abdominal pain. She complains of worsening global weakness and dizziness throughout the encounter as she develops hemorrhagic shock from intra-abdominal bleeding. Students are expected to perform an appropriate primary and secondary survey, initiate needed volume resuscitation, request focused abdominal sonography, and consult surgery.

Case 3: Poly-substance overdose. In this manikin scenario, the patient presents unconscious and is not able to give a history. Examination reveals empty pill and liquor bottles in his pockets. The patient becomes progressively less responsive during the encounter and develops hypoxemia due to airway obstruction and respiratory depression. Students are expected to perform needed assessment, order appropriate diagnostics for altered mental status, and provide airway and respiratory management as hypoxia worsens.

Prior to the OSCE, the students receive a standardized orientation to exam procedures and logistics. During the course of the EM clerkship, students have approximately 20 hours of instructional time, all of which is simulation-based. They are therefore very familiar with simulation and comfortable in the simulation environment prior to the exam.

Following a brief introduction to the patient’s presenting complaint, the student enters and begins the case. Each station includes an in-room confederate playing the role of the patient’s nurse. The confederate roles are extensively scripted to ensure standardization. Confederates are permitted to assist students with locating equipment, obtaining clinical data, administering medications, and performing limited clinical interventions (e.g., chest compressions). Students must otherwise be self-sufficient, as confederates are not permitted to offer suggestions about diagnosis or treatment, and are not permitted to perform clinical interventions outside of their limited scope. Confederates do provide standardized prompts to ensure that the case proceeds in an expeditious fashion, though actions that are prompted by confederates are not given credit on the scoring checklist. The confederates are usually EM educators, though may occasionally be portrayed by simulation center staff members.

All stations are 10 minutes in length with an additional two minutes provided for student feedback. A simulation center staff member controls the timing of the examination. Students receive brief feedback on their performance from the observing faculty member and standardized patient following each case. To ensure psychological safety of the learners, case conclusions are standardized, with all patients stabilized by the end of the scenario. Confederate prompting is designed to ensure that students complete all “life and death” actions, enabling successful resuscitation of the patient before the scenario ends.

Following each station, students are graded using a structured checklist completed in real time by an observing EM faculty member. The standardized patient also completes a checklist to evaluate the students on history-taking and interpersonal skills. Each checklist ranges in length from 29–37 items. The OSCE score is determined by calculating percent of checklist items completed correctly for each station. Station percentages are then averaged to determine the final score, in order to ensure that all stations are weighted equally. For exam security reasons, we are not able to append the final checklists, though we would be happy to privately share our test materials with other educators.

## IMPACT/EFFECTIVENESS

During the first eight months of its administration, the OSCE was used as a pilot test and the students’ performance did not count towards their final grade in the clerkship. Following the pilot period, we analyzed the OSCE data and revised the exam and curriculum accordingly. Currently, performance on the OSCE represents 20% of the student’s final clerkship grade. Other grade components include daily clinical evaluations (55%), a direct observation session (5%), and an internally developed written exam (20%). While we have labored to make our OSCE as psychometrically sound as possible, we recognize the inherent reliability limitations of an exam with a small number of stations. We therefore elected to make it a relatively small part of the students’ final grades.

The OSCE pilot period included a convenience sample of 80 students, all of whose performance data was analyzed. The average score on the exam was 70.5%, with a standard deviation of 7.2%. Scores ranged from 39.3 to 84.1%, and grade distribution data are detailed in the Figure. Of note, the OSCE offers the widest grade distribution of any assessment method used in our clerkship, allowing us to effectively discern students who excel from those who struggle.

Item analysis was completed for all 96 checklist items, including difficulty and discrimination values. Average item difficulty was 70.0%, which is in the “medium” range and is considered appropriate. Average point biserial correlation (r_pb_) was 0.24, which is in the “fair” range, and is considered acceptable though not ideal.

Item difficulty results are presented in Table 2. While there are no universal definitions of item difficulty in educational research, the cutoffs we selected are common. High-difficulty items may suggest a problem with the case, in that it does not provide sufficient clinical clues to prompt students to complete desired actions, or with the items themselves, in that they are not clinically relevant to the case as presented. These items may also reflect a problem with the curriculum, in that the desired action is not being adequately taught. High-difficulty items should not form the foundation on an exam, but some of these items are necessary and desirable for differentiating between low- and high-performing students, as it would be expected that only top students would get these items correct. Likewise, low-difficulty items should also not be overrepresented on an exam, but some of these items are appropriate for the documentation of critical, foundational competencies that every student is expected to know.

Item discrimination results are presented in Table 3. Poor discrimination means that overall low-performing learners get an item correct, while high performers get the item incorrect. Easy items will always discriminate poorly, as they are completed correctly by low-performers and high-performers alike. As noted above, this is not always problematic, particularly for items that reflect universal basic competencies, like initiating chest compressions for cardiac arrest. However, poor discrimination may also suggest a problem with clinical aspects of the case or item.

When completing an item analysis, difficulty and discrimination must be considered simultaneously, and item revision decisions are never made based solely on one or the other. For example, a high-difficulty item might be retained if it discriminates effectively between students. It is also essential to consider each item in the context of its purpose in the exam. For example, poor discrimination is acceptable for a foundational item that is expected to be “easy.” However, it is problematic for an item that is intended to be difficult, as this means that strong and weak students are equally likely to miss the item, suggesting that it is either not taught in the curriculum or not adequately cued by the case.

Based on our item analysis, we removed 11 items. Of these, half were high-difficulty items that were poor discriminators, in most cases because they were too challenging for even strong students to complete in the very limited time available. The other half were low-difficulty items with poor discrimination that were not felt to be sufficiently foundational to remain on the list. We revised the case and/or item to address concerns about 13 items. Most revisions were made for high-difficulty items in which case was adjusted to make the need for the action in question more obvious. There were also six items that led to curricular adjustments in order to emphasize key points and teach key concepts more effectively.

## LIMITATIONS

There are, of course, important limitations to address. First and foremost, this project was conducted at a single site, and the results may not generalize to other institutions. Second, we present only preliminary pilot data, though we are currently in process of implementing our revised exam with another group of students and will be able to determine whether our revisions improve the psychometric performance of the exam. Last and most important was our inability to fully validate the exam. Doing this would require development of a true “gold standard” against which to compare student performance on this assessment. No such standard currently exists, particularly for clinical performance. Given the very significant limitations of “real” clinical assessment, attainment of this standard may prove elusive.

That said, we believe that implementation of our OSCE has provided us with valuable information regarding the performance of our students at the completion of their basic clerkship. It gave us a unique window on their ability to independently evaluate and manage acutely ill and injured patients, as it required them to apply knowledge and skills gained throughout the course of the rotation to “real” clinical problems. We learned what things the students reliably do well, and where they struggle. We also identified common errors and misconceptions, enabling us to strengthen our teaching in these areas. This examination can easily be adapted at other institutions provided they have access to simulation technology. Our cases test core EM content reflected in clinically-oriented Milestones, and allow assessment of the student’s ability to manage these issues independently in a way that would be impossible in the clinical area.

## CONCLUSION

Overall, we found that the OSCE effectively discriminates between high- and low-performing students in a way that other assessment tools do not. The score range on the OSCE is wider than that of our written exam, and our clinical evaluations (like those of many institutions) suffer from a severe restriction of range that limits their utility in differentiating between students – a problem we do not see with the OSCE. The OSCE also offers insight into aspects of student performance that are not captured through other means of evaluation. Overall, we believe that OSCEs offer a useful tool for assessment of EM knowledge and skills, and they can provide a foundation for documentation of the essential competencies reflected in the Milestones and the newer Entrustable Professional Activities.^17^

## Figures and Tables

**Figure:**
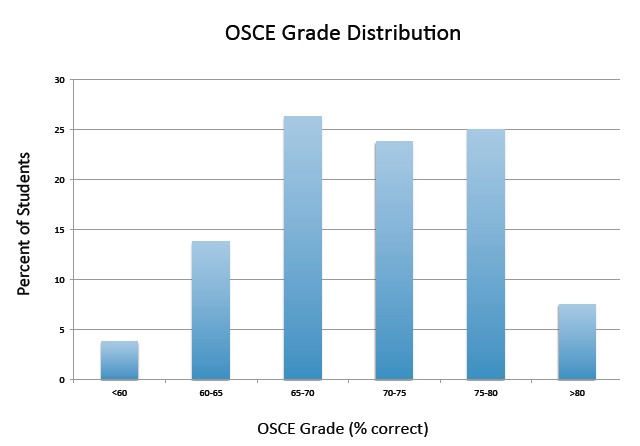
Objective structured clinical exam (OSCE) grade distribution. Each bar represents the percent of students achieving a final score within the grade range listed on the X-axis.

**Table 1:** Mapping checklist items to emergency medicine milestones.

Milestone	Description	Case(s)	Checklist items
1	Emergency stabilization	All	- Recognition of abnormal vital signs - Primary assessment on critically injured patient
2	Performance of focused history and physical exam	All	- Obtains focused history - Obtains focused physical examination
3	Diagnostic studies	All	- Requests CXR and ECG - Considers Chest CT - Requests appropriate laboratory testing (including acetaminophen level, alcohol level, d-dimer)
4	Diagnosis	All	- Considers pulmonary embolus in diagnosis - Considers intra-abdominal bleeding in diagnosis - Considers acetaminophen, opioid, alcohol overdose
5	Pharmacotherapy	All	- Requests and administers rapid sequence intubation medications - Administers epinephrine in cardiac arrest - Requests N-acetyl cysteine for treatment of acetaminophen overdose
6	Observation and reassessment	All	- Reassesses vital signs after return of spontaneous circulation following cardiac arrest - Reassesses vital signs after administration of intravenous fluids
10	Airway management	3	- Effectively bag valve masks patient and troubleshoots BVM technique - Performs rapid sequence intubation in patient with airway compromise
12	Goal-directed focused ultrasound	2	- Orders FAST exam in patient with abnormal vital signs following trauma
20	Professional values	2	- Standardized patient assesses student’s interpersonal skills - Demonstrates behavior that conveys caring, honesty and genuine interest and tolerance
22	Patient centered care	2	- Standardized patient assesses student’s interpersonal skills - Establishes rapport and demonstrates empathy towards patient - Effectively listens to patient

*CXR,* chest x-ray; *ECG*, electrocardiogram; *CT*, computed tomography; *FAST*, focused assessment with sonography for trauma

**Table 2:** Item difficulty of OSCE components.

Difficulty level	Number of checklist items
Low (>80% correct)	36 (37%)
Medium (50–80% correct)	41 (43%)
High (<50% correct)	19 (20%)

**Table 3:** Item discrimination of OSCE components.

Discrimination level	Number of checklist items
Good (r_pb_>0.3)	10 (11%)
Fair (r_pb_=0.1–0.3)	47 (51%)
Poor (r_pb_<0.1)	35 (38%)
